# Short video platforms as sources of health information about cervical cancer: A content and quality analysis

**DOI:** 10.1371/journal.pone.0300180

**Published:** 2024-03-08

**Authors:** Juanjuan Zhang, Jun Yuan, Danqin Zhang, Yi Yang, Chaoyun Wang, Zhiqian Dou, Yan Li

**Affiliations:** 1 Center for Reproductive Medicine, Taihe Hospital, Hubei University of Medicine, Shiyan, Hubei, China; 2 Department of Ophthalmology, Taihe Hospital, Hubei University of Medicine, Shiyan, Hubei, China; 3 Department of Gastroenterology, Taihe Hospital, Hubei University of Medicine, Shiyan, Hubei, China; 4 Department of Gynaecology and Obstetrics, Taihe Hospital, Hubei University of Medicine, Shiyan, Hubei, China; Ege University, Faculty of Medicine, TURKEY

## Abstract

**Background:**

The development of short popular science video platforms helps people obtain health information, but no research has evaluated the information characteristics and quality of short videos related to cervical cancer. The purpose of this study was to evaluate the quality and reliability of short cervical cancer-related videos on TikTok and Kwai.

**Methods:**

The Chinese keyword "cervical cancer" was used to search for related videos on TikTok and Kwai, and a total of 163 videos were ultimately included. The overall quality of these videos was evaluated by the Global Quality Score (GQS) and the modified DISCERN tool.

**Results:**

A total of 163 videos were included in this study, TikTok and Kwai contributed 82 and 81 videos, respectively. Overall, these videos received much attention; the median number of likes received was 1360 (403–6867), the median number of comments was 147 (40–601), and the median number of collections was 282 (71–1296). In terms of video content, the etiology of cervical cancer was the most frequently discussed topic. Short videos posted on TikTok received more attention than did those posted on Kwai, and the GQS and DISCERN score of videos posted on TikTok were significantly better than those of videos posted on Kwai. In addition, the videos posted by specialists were of the highest quality, with a GQS and DISCERN score of 3 (2–3) and 2 (2–3), respectively. Correlation analysis showed that GQS was significantly correlated with the modified DISCERN scores (p<0.001).

**Conclusion:**

In conclusion, the quality and reliability of cervical cancer-related health information provided by short videos were unsatisfactory, and the quality of the videos posted on TikTok was better than that of videos posted on Kwai. Compared with those posted by individual users, short videos posted by specialists provided higher-quality health information.

## Introduction

Cervical cancer is the fourth most common cancer among women worldwide, with an estimated 604,000 new cases in 2020 [1]. Chronic infection with human papillomavirus (HPV) leads to almost all cases of cervical cancer [2], and other risk factors include early age at sexual debut, having multiple sexual partners or a high-risk sexual partner, and immunosuppression [3]. Due to the lack of organized screening and human papilloma virus (HPV) vaccination programs, approximately 90% of cervical cancer cases occur in low-income and middle-income countries [3]. A previous study showed that the 5-year survival rate of patients with early cervical cancer after radical hysterectomy was 87% [4], while that of patients with advanced cervical cancer was only 28.4% [5]. Despite cervical cancer causing serious damage to human health, many studies have shown a general lack of health knowledge about cervical cancer [6, 7]. Therefore, it is urgent to improve the level of awareness of cervical cancer among women through effective information and health education.

Social media is increasingly used in public health education because of its ability to remove physical barriers that have traditionally prevented access to health care support and resources [8]. The public and health professionals can communicate about health issues through social media, potentially improving health outcomes [9]. A previous study showed that health information dissemination can change health behaviors, thus greatly improving the early diagnosis and prevention of diseases [10]. However, some nonprofessionals and even patients may use social media to communicate and share information, which may lead to the spread of misinformation [11]. A meta-analysis revealed that considerable misinformation on major public health issues, such as smoking, drugs, and disease, was available on social media [12]. As an emerging social communication medium, short video sharing platforms have become popular among the general public. However, no study has evaluated the quality of videos about cervical cancer on short video sharing platforms. Therefore, the purpose of this research was to evaluate the information quality and reliability of videos related to cervical cancer from TikTok and Kwai.

## Materials and methods

### Data collection

The Chinese keyword "cervical cancer" was used to search related videos on two short video sharing platforms (TikTok and Kwai) in China, and the top 100 videos were screened according to the default comprehensive ranking. The videos were reviewed by two obstetricians and gynaecologists (Wang C and Dou Z), and the following videos were excluded: repeated videos, silent or poor sound quality videos, adverts, and videos unrelated to the topic. Two researchers reviewed the video independently and recorded the data in Excel (Microsoft Corporation). Data collection included video source, the name and identity authentication of the uploader, publication date, departments of health professionals, video duration, the number of likes, comments and collections, video content, and the quality of the video (**S1 Appendix**). The data used in this study were obtained from TikTok (https://www.douyin.com) and Kwai (https://www.kuaishou.com). This study did not involve human or animal subjects and therefore did not require ethical review.

### Related methods and definitions

Prior to evaluating the videos, two independent investigators reviewed the cervical cancer-related guidelines, Global Quality Score (GQS), and modified DISCERN tool. The GQS is a widely used tool for evaluating the quality of health information provided in videos [13]. The researchers evaluated the quality of videos and their benefits to patients and assigned them a score ranging from 1 to 5 according to quality (very poor to very good). The reliable and valid DISCERN tool was first used for judging the quality of written consumer health information [14]. The modified DISCERN tool was used to assess the reliability of video content [15]. When reviewing a video, the researcher evaluated whether the video met the following standards: clarity, relevance, traceability, robustness and fairness. The above questions were answered yes (1 point) or no (0 points), and the cumulative score was calculated (0–5 points). In addition, the completeness score of the video was evaluated according to whether the video included the following information: epidemiology, etiology, symptoms, diagnosis, treatment, prevention, and prognosis (**S2 Appendix**). The videos were graded as not explained (0 points), partially explained (1 point) or fully explained (2 points) according to whether the video uploader clearly explained the content of the related topic. For example, when a video’s explanation of the etiology of cervical cancer met more than three-quarters of the content list, we gave it a score of 2 points (fully explained). If the video did not mention this, a score of 0 was assigned. Specialists included obstetricians, gynecologists and oncologists, and nonspecialists mainly included dermatologists, traditional Chinese medicine doctors and radiologists.

### Statistical analysis

Categorical variables are expressed as frequencies and percentages, and chi-square tests or Fisher’s exact tests were performed as appropriate. For data following a normal distribution, continuous variables were presented as the mean ± standard deviation (SD), and data were analyzed by using Student’s t-test. For data that did not follow a normal distribution, the continuous variables were represented in the form of median and interquartile intervals (IQR), and the Mann-Whitney U test was used to analyze the data. To assess the agreement of the ratings between the two reviewers, Cohen kappa coefficients were calculated. The Spearman test was used to evaluate the correlation between different scores and video features. All the statistical analyses were performed with R statistical software version 4.3.1 (www.r-project.org), and a two-tailed P value < 0.05 was considered to indicate statistical significance.

## Results

### Videos characteristics

**Fig 1:**
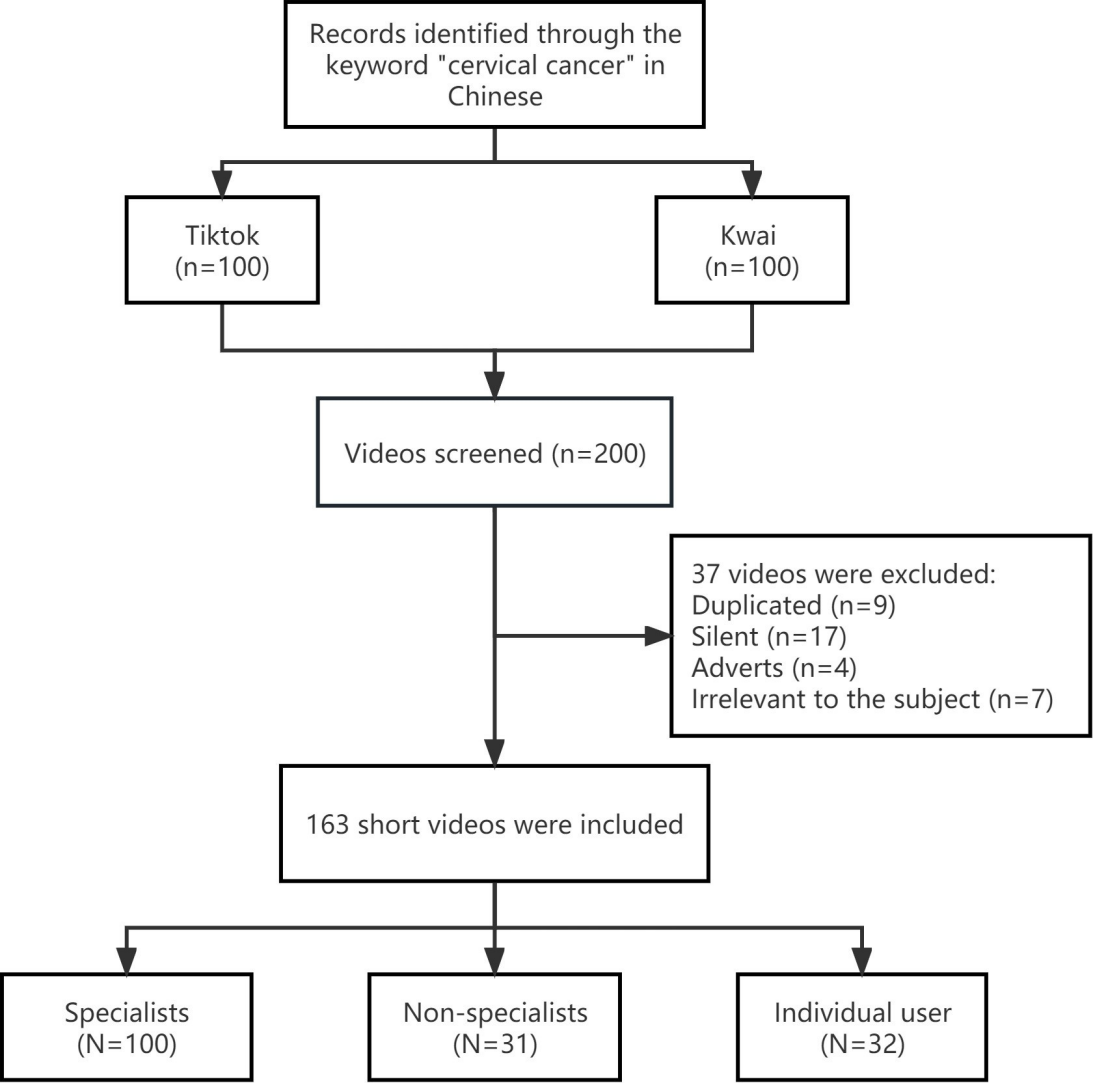
The flow chart of this study.

According to the inclusion and exclusion criteria, the top 100 videos on TikTok and Kwai were screened, and a total of 163 videos were ultimately included in this study (**Fig 1**). **Table 1** presents the characteristics of the included videos. A total of 82 and 81 videos were obtained from TikTok and Kwai, respectively. Among the videos, specialists posted the most videos, accounting for 61.4%, while nonspecialists and individual users posted 19.0% and 19.6% of the videos, respectively. Overall, these videos received much attention; the median number of likes received was 1360 (403–6867), the median number of comments was 147 (40–601), and the median number of collections was 282 (71–1296). The length of these videos was shorter, with a median time of 74 (47–109) seconds, and the completeness score was 3 (2–4). The median GQS of these videos was 3 (2–3), and the median DISCERN score was 2 (1–3). The Cohen kappa values of the GQS and modified DISCERN score were 0.914 and 0.901, respectively. These results were in the range of 0.81–1, indicating good agreement between the scores of the two independent reviewers.

**Table 1:** Video characteristics.

Characteristic		N = 163
Short-video sharing platforms [n (%)]		
	TikTok	82 (50.3)
	Kwai	81 (49.7)
Video source [n (%)]		
	Specialists	100 (61.4)
	Non-specialists	31 (19.0)
	Individual user	32 (19.6)
Number of likes [median (IQR)]		1360 (403–6867)
Number of comments [median (IQR)]		147 (40–601)
Number of collections [median (IQR)]		282 (71–1296)
Video duration [s, median (IQR)]		74 (47–109)
Completeness score [median (IQR)]		3 (2–4)
GQS scores [median (IQR)]		3 (2–3)
DISCERN scores [median (IQR)]		2 (1–3)

### Video content

We further analyzed the content discussed in the included short videos (**Table 2**). We found that the etiology of cervical cancer was the most frequently discussed topic, with 30% of the videos providing a full explanation of it. In addition, the symptoms and diagnosis of cervical cancer were also topics of public concern, with 20.2% and 15.9% of the videos providing detailed explanations, respectively. However, few videos provide detailed health information on the epidemiology and prognosis of cervical cancer.

**Table 2:** Completeness of video content.

Video content	Not involve (0 points)	Partial explanation (1 point)	Full explanation (2 points)
Epidemiology, n (%)	131 (80.4)	26 (16.0)	6 (3.6)
Etiology, n (%)	79 (48.5)	35 (21.5)	49 (30.0)
Symptoms, n (%)	115 (70.6)	15 (9.2)	33 (20.2)
Diagnosis, n (%)	123 (75.5)	14 (8.6)	26 (15.9)
Treatment, n (%)	111 (68.1)	33 (20.2)	19 (11.7)
Prevention, n (%)	115 (70.6)	28 (17.2)	20 (12.2)
Prognosis, n (%)	141 (86.5)	10 (6.1)	12 (7.4)

### Characteristics comparison of different platforms

**Fig 2:**
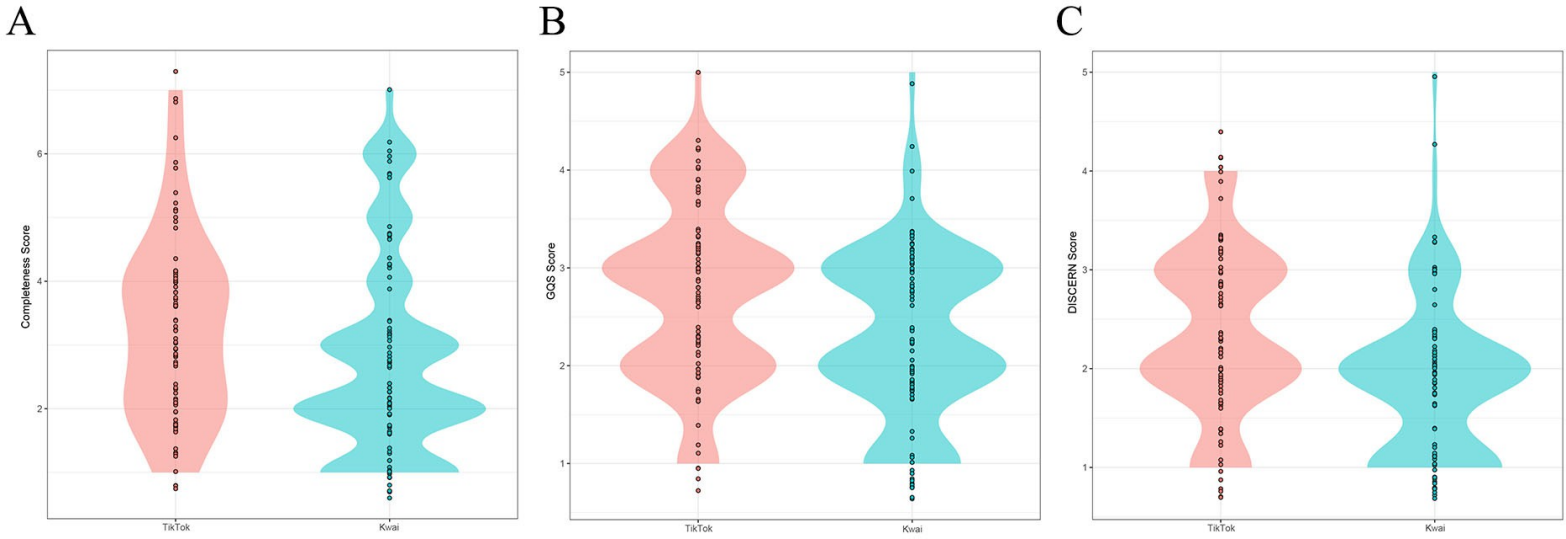
Comparison of of videos from different platforms. A: Completeness score; B: GQS score; C: Modified DISCERN score.

**Table 3** shows the video characteristics and quality of the different short video sharing platforms. The video sources of Tiktok and Kwai are obviously different. A total of 74.4% of the videos posted on TikTok were uploaded by specialists, while only 48.8% of the videos posted on Kwai were uploaded by specialists. The number of likes, comments and collections of videos on TikTok were 2849 (401–13250), 375 (63–985) and 410 (73–1998), respectively. The number of likes, comments and collections of videos in Kwai were 852 (374–3593), 102 (36–274) and 176 (71–1060), respectively. The median completeness score of videos posted on TikTok was 3 (2–4), which was significantly greater than that of videos posted on Kwai. In terms of video quality, the GQS and DISCERN score of videos posted on TikTok were significantly better than those of videos posted on Kwai (**Fig 2**).

**Table 3:** Comparison of different short-video sharing platforms.

Variables	TikTok (N = 82)	Kwai (N = 81)	p valve
Video source [n (%)]			0.001
Specialists	61 (74.4)	39 (48.1)	
Non-specialists	8 (9.8)	23 (28.4)	
Individual user	13 (15.9)	19 (23.5)	
Number of likes [median (IQR)]	2849 (401–13250)	852 (374–3593)	0.015
Number of comments [median (IQR)]	375 (63–985)	102 (36–274)	0.006
Number of collections [median (IQR)]	410 (73–1998)	176 (71–1060)	0.015
Completeness score [median (IQR)]	3 (2–4)	2 (2–3.5)	0.016
GQS scores [median (IQR)]	3 (2–3)	2 (2–3)	0.006
DISCERN scores [median (IQR)]	2 (2–3)	2 (1–2)	<0.001

### Characteristics comparison of different video sources

**Fig 3:**
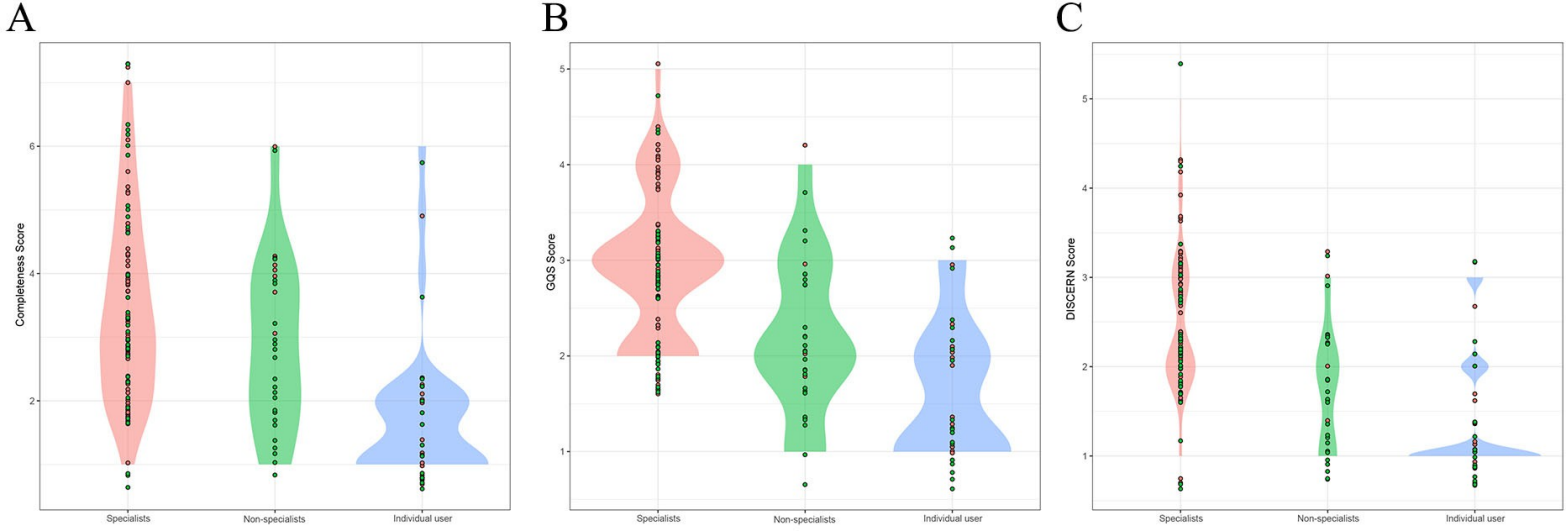
Comparison of of videos from different sources. A: Completeness score; B: GQS score; C: Modified DISCERN score.

We further compared the characteristics and quality of short videos from different sources. In terms of video popularity, there was no significant difference in the number of likes, comments or collections of videos posted by specialists, nonspecialists or individual users (**Table 4**). The video completeness scores of specialists and nonspecialists were 3 (2–4) and 3 (2–4), respectively, which were significantly greater than the score of 1 (1–2) for videos posted by individual users. In addition, the videos released by specialists were of the highest quality, with a GQS and DISCERN score of 3 (2–3) and 2 (2–3), respectively. However, the quality of videos released by individual users was generally poor (**Fig 3**).

**Table 4:** Comparison of different video source.

Variables	Specialists (N = 100)	Non-specialists (N = 31)	Individual user (N = 32)	p valve
Number of likes [median (IQR)]	1998 (414–8052)	736 (201–3995)	1610 (606–4398)	0.213
Number of comments [median (IQR)]	158 (42–597)	125 (18–226)	245 (39–1459)	0.347
Number of collections [median (IQR)]	369 (97–1474)	239 (26–1385)	170 (40–645)	0.564
Completeness score [median (IQR)]	3 (2–4)	3 (2–4)	1 (1–2)	0.003
GQS scores [median (IQR)]	3 (2–3)	2 (2–3)	1 (1–2)	0.010
DISCERN scores [median (IQR)]	2 (2–3)	2 (1–2)	1 (1–2)	<0.001

### Correlation analysis between different scores and video features

**Table 5** shows the correlation analysis between different scores and video features. There was a significant correlation between the GQS and modified DISCERN score (p<0.001). There was a positive correlation between the duration and number of collections of videos and the GQS. In addition, there was a correlation between the number of video collections and the modified DISCERN score (p = 0.013).

**Table 5:** Correlation of GQS and modified DISCERN scores with video features.

	GQS		Modified DISCERN	
	r	p valve	r	p valve
GQS	-	-	0.839	<0.001
Modified DISCERN	0.839	<0.001	-	-
Likes	0.128	0.104	0.137	0.081
Comments	0.057	0.466	0.084	0.285
Collections	0.185	0.018	0.194	0.013
Video duration	0.206	0.008	0.12	0.127

## Discussion

Social media has been widely used in the field of health, and the rapid development of social media provides opportunities for better health communication and patient education [16]. As two of the most popular short video platforms in China, TikTok and Kwai have gained great popularity among ordinary health consumers [17, 18]. On the one hand, short video applications provide a platform for consumers to quickly obtain health information; on the other hand, short videos can obtain large amounts of traffic and attention. However, the misinformation in some posted videos can also spread quickly and affect the health of the general public [19]. Therefore, we designed this study to assess the quality and reliability of cervical cancer-related health information in videos posted on TikTok and Kwai.

In this study, we found that short cervical cancer-related videos received much attention, with four videos receiving more than 100,000 likes. One of the short videos posted by a cardiologist provided a detailed explanation of the epidemiology, etiology and prevention of cervical cancer; this video had 198,000 likes, 11,000 comments and 50,000 collections. For health information about the prevention of cervical cancer, which is of greater concern to the public, only a few videos discussed this topic. Although the vast majority of professionals mentioned HPV vaccination as an effective measure for cervical cancer prevention, we found that a patient provided incorrect information that the HPV vaccine is ineffective, which is bound to cause distress for the general public, which lacks relevant expertise. The rapid dissemination of high-quality videos is beneficial for the health education of ordinary people [20]. However, our results suggest that the quality and reliability of health information about cervical cancer available on short video sharing platforms are unsatisfactory and that the quality of videos posted by different creators is significantly different. Among the videos, the quality and reliability of the videos posted by specialists were significantly better than that of videos posted by individual users because of their sufficient professional knowledge. Previous studies have shown similar results [21, 22]. Therefore, more specialists are encouraged to promote health education for all people by sharing health education videos [23]. In addition, we evaluated the quality of short videos on different platforms, and the information quality of short videos from TikTok was better than that of short videos from Kwai. This may be due to the varying levels of regulation of health-related videos on different platforms. On TikTok, the vast majority of health education videos was posted by specialists, while Kwai had a greater proportion of videos posted by individual users. The quality of videos posted by individual users is significantly worse due to the lack of a relevant professional background. Health education videos are different from other videos, as the spread of incorrect information may misinform people about diseases and even harm their health. A previous study revealed that many videos spread misinformation that anorexia is a healthy lifestyle, and these videos are very popular among viewers [24]. Therefore, strengthening the platform’s supervision of posted health education videos is crucial.

To the best of our knowledge, this is the first study to evaluate cervical cancer-related health information on short video platforms. This study has several limitations. First, this study evaluated only short videos posted on TikTok and Kwai, and the information quality of other platforms still needs further research. Second, the data for short cervical cancer-related videos may change as some videos are uploaded and deleted. Third, this study included only short videos in Chinese, and short videos in other languages still need to be evaluated.

In conclusion, the quality and reliability of the cervical cancer-related health information provided by short videos were unsatisfactory, and the quality of the videos posted on TikTok was better than that of videos posted on Kwai. Compared with those posted by individual users, short videos posted by specialists can provide higher-quality health information.

## Supporting information

S1 Appendix Original data set used for the current study. (XLSX)

S2 Appendix Lists of training content. (DOCX)
