# Supplementary material for: Short video platforms as sources of health information about cervical cancer: A content and quality analysis
Source: PLoS One. 2024 Mar 8;19(3):e0300180. doi: 10.1371/journal.pone.0300180 (PMC10923403; doi:10.1371/journal.pone.0300180)
Supplement: S2 Appendix — (DOCX) [file pone.0300180.s002.docx]

**Lists of training content**

**1. Epidemiology**

1) Global morbidity

2) Global mortality

3) Morbidity in China

4) Mortality in China

**2. Etiology**

1) HPV infection

2) Early age of sexual debut

3) Multiple sexual partners or a high-risk sexual partner

4) Immunosuppression

5) History of sexually-transmitted infection

**3. Symptoms**

1) Early stages: often asymptomatic

2) Abnormal vaginal bleeding

3) Profuse malodorous vaginal discharge

4) Symptoms of invasion of other organs

**4. Diagnosis**

1) Cervical cytology and HPV test

2) Colposcopy

3) Gynecological examination

4) Histopathological assessment of a cervical biopsy

5) Imageological examination

**5. Treatment**

1) Surgical treatment

2) Radiotherapy

3) Chemotherapy

4) Immunotherapy

**6. Prevention**

1) HPV vaccination

2) Abstaining from sexual activity

3) Mutual monogamy of virgins

4) The use of condoms

5) Secondary prevention.

**7. Prognosis**

1) Prognosis of different stages

2) Risk factors affecting prognosis
